# Fine needle aspirate flow cytometric phenotyping characterizes immunosuppressive nature of the mesothelioma microenvironment

**DOI:** 10.1038/srep31745

**Published:** 2016-08-19

**Authors:** Patrick H. Lizotte, Robert E. Jones, Lauren Keogh, Elena Ivanova, Hongye Liu, Mark M. Awad, Peter S. Hammerman, Ritu R. Gill, William G. Richards, David A. Barbie, Adam J. Bass, Raphael Bueno, Jessie M. English, Mark Bittinger, Kwok-Kin Wong

**Affiliations:** 1 Belfer Center for Applied Cancer Science, 360 Longwood Ave. Boston, MA 02115, USA; 2 Department of Medical Oncology, Dana-Farber Cancer Institute, 450 Brookline Ave, Boston, MA 02115, USA; 3 Lowe Center for Thoracic Oncology, Dana-Farber Cancer Institute, 450 Brookline Ave, Boston, MA 02115, USA; 4 Harvard Medical School, 25 Shattuck St, Boston, MA 02115, USA; 5 Department of Radiology, Brigham and Women’s Hospital, 75 Francis St. Boston, MA 02115, USA; 6 Division of Thoracic Surgery, Brigham and Women’s Hospital, 75 Francis St. Boston, MA 02115, USA; 7 International Mesothelioma Program of the Lung Center Surgery at Brigham and Women’s Hospital, 75 Francis St. Boston, MA 02115, USA.

## Abstract

With the emergence of checkpoint blockade and other immunotherapeutic drugs, and the growing adoption of smaller, more flexible adaptive clinical trial designs, there is an unmet need to develop diagnostics that can rapidly immunophenotype patient tumors. The ability to longitudinally profile the tumor immune infiltrate in response to immunotherapy also presents a window of opportunity to illuminate mechanisms of resistance. We have developed a fine needle aspirate biopsy (FNA) platform to perform immune profiling on thoracic malignancies. Matching peripheral blood, bulk resected tumor, and FNA were analyzed from 13 mesothelioma patients. FNA samples yielded greater numbers of viable cells when compared to core needle biopsies. Cell numbers were adequate to perform flow cytometric analyses on T cell lineage, T cell activation and inhibitory receptor expression, and myeloid immunosuppressive checkpoint markers. FNA samples were representative of the tumor as a whole as assessed by head-to-head comparison to single cell suspensions of dissociated whole tumor. Parallel analysis of matched patient blood enabled us to establish quality assurance criteria to determine the accuracy of FNA procedures to sample tumor tissue. FNA biopsies provide a diagnostic to rapidly phenotype the tumor immune microenvironment that may be of great relevance to clinical trials.

There is an urgent unmet need to understand the mechanisms of innate and acquired resistance to immunotherapy. Antibodies that block immunosuppressive cell surface molecules such as PD-1, PD-L1, PD-L2, CTLA-4, LAG-3, TIM-3, and VISTA^1^,^2^,^3^,^4^,^5^ or act as agonists to activate effector lymphocyte co-receptors 4-1BB, OX40, and GITR^6^,^7^ are currently in clinical development or are already approved for use in patients. The ability to profile the tumor immune infiltrate before, during, and after treatment with immunomodulatory agents is needed to improve patient care and the selection of appropriate immunotherapies for a given patient, and will advance our understanding of the complex cellular interactions occurring in the tumor microenvironment in response to these therapies.

Collection of guided fine needle aspirates (FNA) from tumors is a common clinical procedure that has been routinely utilized during the past 15–20 years. FNA have been shown to yield more viable cells than needle core biopsies, allowing for generation of single cell suspensions amenable to immediate flow cytometric analysis^8^. For this reason, they have been used with multiparametric flow cytometry as a method to augment the diagnosis of lymphoproliferative disorders, principally lymphomas, and report high concordance (over 95%) with conventional histopathological examination, which is considered the most rigorous methodology^9^,^10^,^11^. Although a study from 2004 suggested poor correlation between FNA and excisional biopsies for lymphoma^12^, a more recent study by the American Society of Cytopathology demonstrated that FNA do, in fact, recapitulate findings made from excisional biopsies^13^. While FNA are a reliable method for diagnosis, this technique has not been used to determine the immunosuppressive phenotype of the tumor microenvironment in established cancers.

Biopsies such as FNA are relevant for clinical trials because they enable longitudinal tracking that can identify patients who may benefit from this new class of immunotherapeutics and, importantly, can also be used to identify and study patients who do not respond to treatment. For instance, immunohistochemical determination of PD-L1 expression is technically challenging and, in general, pathological scoring is impacted by tumor heterogeneity and displays only moderate correlation with response to PD-1/PD-L1 blockade clinically^14^. Compared to IHC, flow cytometry is uniquely capable of quantitatively determining leukocyte abundance and expression levels of numerous immune checkpoint and co-stimulatory markers.

FNA biopsies are thought to be more cellular compared with core biopsies and, therefore, may yield sufficient material for comprehensive analysis with multiple antibody panels; however, it is not known whether they accurately represent the immunophenotype of the whole tumor in question. We sought to develop a FNA analysis platform to perform immune profiling of biopsy samples, to assess concordance with other immune profiling methods, and to determine the feasibility of implementing this technique for diagnostic use in clinical trials. We hypothesize that tumor FNA will yield adequate tissue to perform in-depth immunoprofiling and that FNA will recapitulate the immunophenotypic profile of the bulk resected tumor. Phenotypic analyses were performed on FNA from 13 patients with Malignant Pleural Mesothelioma.

## Results

### Fine needle aspirates are superior to core needle biopsies

**Figure 1:**
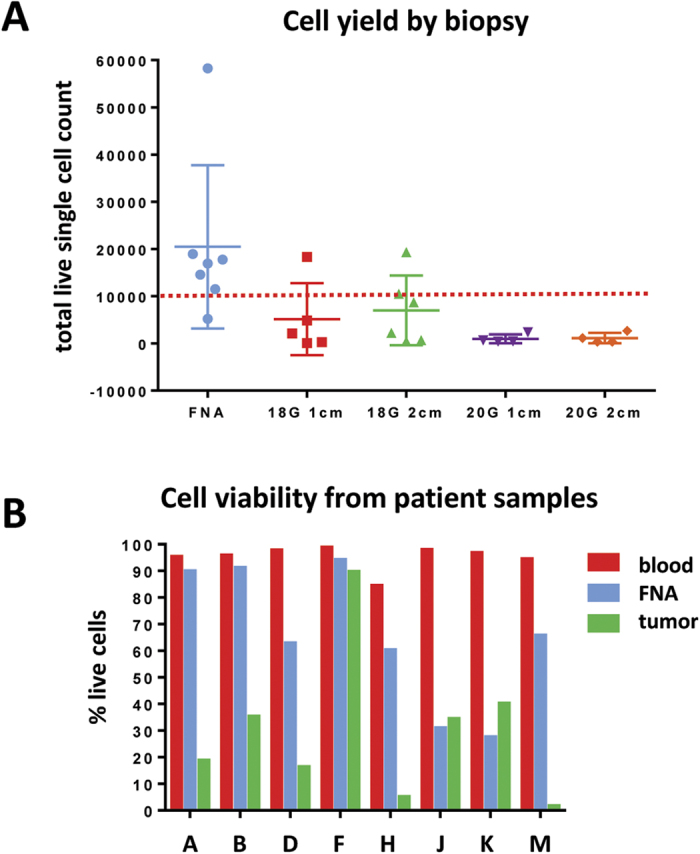
Fine needle aspirate biopsies yield high numbers of viable cells. (**A**) NSCLC FNA biopsies consistently collect more viable cells relative to other core needle biopsy techniques. (**B**) Mesothelioma FNA also yield a higher percentage of viable cells compared to dissociated resected tumors (**C**).

We first assessed seven non-small cell lung cancer (NSCLC) patients who were undergoing surgical resection and consented to an IRB-approved tissue collection protocol. For each patient, the excised tumors were subjected *ex vivo* to FNA and multiple core biopsies to determine cellularity. Fine needle aspirates (FNA) provided greater numbers of viable cells when compared to 18 G and 20 G core biopsies at both 1 cm and 2 cm lengths (Fig. 1A) and FNAs provided over 10,000 viable cells in 6/7 samples with a mean of 20,455 and SEM of 6,544. 10,000 viable cells provided sufficient material to run multiple flow cytometry antibody panels (Fig. 1A red dashed line; Supplementary Figure 2). FNA samples did not require enzymatic dissociation for flow cytometry, a procedure associated with a loss in viability of tumor and immune cells. Samples were also processed fresh and not freeze/thawed, which is known to preferentially kill CD45^−^ cells and granulocytes and enrich for lymphocytes (Supplementary Figure 3). Therefore, the FNA collection generated cells that could be immediately analyzed, without excessive loss of viability encountered with the lengthy dissociation as is typically performed with core biopsy specimens.

In order to establish that this technique is applicable *in vivo* via percutaneous FNA, we also collected single pass FNA from 13 patients undergoing surgery for mesothelioma. To better approximate clinical collection of FNA as compared to NSCLC explants, we performed ultrasound-guided FNA of tumors prior to surgical resection in 13 mesothelioma patients. In 8 of the 13 cases, a single-pass FNA sample yielded enough cells for flow cytometry analysis (Supplementary Figure 4). Although FNA from these eight yielded significantly fewer total cells as compared to the resected bulk tumor (Supplementary Figure 4), FNA provided a greater percentage of live cells when compared to analysis of the tumor specimen (Fig. 1B). Resected tumors were without blood supply for at least 30 min prior to this distribution from the frozen section lab, which may account for the reduced viability of cells from whole dissociated tumors.

### FNA are representative of resected tumors

**Figure 2:**
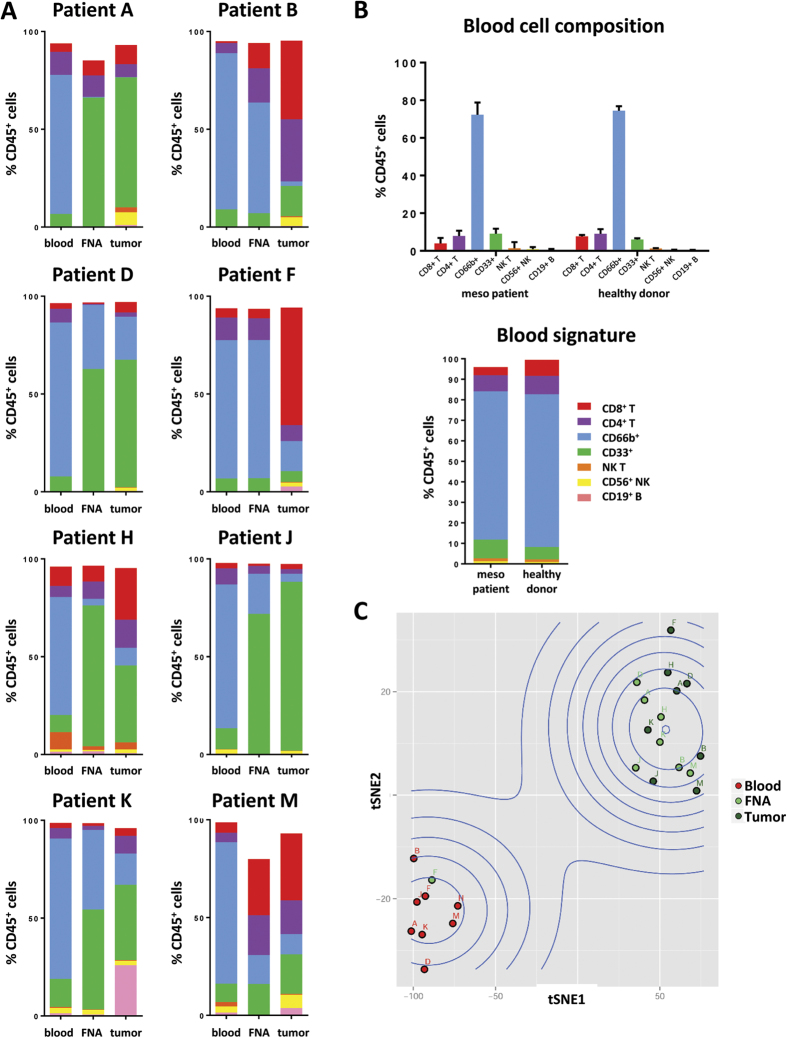
FNA cell composition matches mesothelioma cell composition. (**A**) FNA that yielded suitable numbers of cells were analyzed by flow cytometry along with matching peripheral blood and dissociated whole tumor. (**B**) Peripheral blood samples from multiple mesothelioma patients and healthy donors revealed tight distributions of cell populations (top panel) that enabled construction of a blood signature (bottom panel) as an internal reference control. Top panel plotted as Mean ± SEM with bottom panel comprised of Mean % values. (**C**) Figure shows the samples in the space of t-SNE1 and t-SNE2 with colors labeling the tissue types (Blood = red, FNA = light green, tumor = dark green).

We next sought to determine by detailed flow cytometric profiling whether leukocyte phenotypic markers captured by FNA recapitulated our findings from whole tumor resections. To determine if FNA were representative of the bulk tumor, we generated a dataset of matched blood, FNA, and bulk resected tumor samples from the same cohort of 13 mesothelioma patients mentioned above (Fig. 2). Eight out of thirteen FNA yielded enough cells for comprehensive immune cell phenotyping. Flow cytometric analysis revealed a clear signature for blood samples (given as Mean ± SEM): CD8^+^ T cells 3.99% ± 1.09, CD4^+^ T cells 7.86% ± 1.07, CD33^+^ monocytes 9.16% ± 1.01, CD66b^+^ granulocytes 72.30% ± 2.44, NK cells 2.30% ± 0.42, B cells 1.13% ± 0.24, and NK T cells 2.95% ± 2.00 (Fig. 2B). The leukocyte composition of mesothelioma patient blood matched blood collected from healthy donors (Fig. 2B). Tumor tissues, however, shared little consistency between patients with respect to the proportions of specific immune cell populations present in their associated immune infiltrates. Due to the actual physical distribution of mesothelioma tumors, the tumor specimens utilized for flow cytometry were most likely from different locations in the tumor compared to the FNA sites. Nonetheless, leukocyte population distributions from FNA samples largely aligned with the signatures of their dissociated matching bulk resected tumors (Fig. 2A). An unsupervised, non-linear dimensional reduction method, t-distributed stochastic neighbor embedding (t-SNE), was applied to the eight patients for whom we had full immune profiling from blood, FNA, and tumor. Samples clustered into two clear nodes: the first of which was comprised of the eight blood samples and one FNA sample, and the second with all 8 tumor samples and the remaining seven FNA samples (Fig. 2C). Notably, the FNA sample from patient F did not match the respective tumor profile, but more closely aligned to the respective blood profile, suggesting contamination. Blood samples have highly consistent leukocyte composition between patients (Fig. 2A,B), which enabled us to identify a “blood signature” that is principally defined by the high percentage of CD66b^+^ neutrophils (Fig. 2B). Additionally, paired FNA and tumor samples tended to group together in multidimensional space based on immune profiling parameters, further indicating that FNA recapitulated what we observed from bulk tumors (Fig. 2C).

Our flow cytometry immunophenotyping platform was also highly accurate and reproducible. We observed minimal discrepancy in population distribution and expression of phenotypic markers between technical replicates, even for intracellular transcription factors like FOXP3, which are experimentally more difficult to measure (Supplementary Figure 5). Our process was also unaffected by very low cell input, as highly diluted samples, like FNA, yielded concordant results when compared to more cellular samples from the same tumor (Supplementary Figure 6).

### FNAs characterize immunosuppressive tumor microenvironment

**Figure 3:**
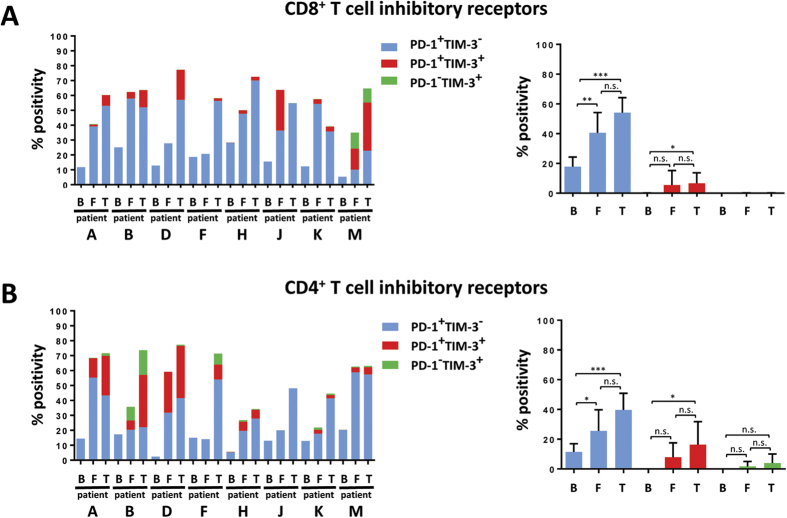
Analysis of mesothelioma FNA by flow cytometry characterizes tumor immunophenotype. (**A,B**) Immunoprofiling revealed high expression of inhibitory receptors on CD8^+^ T cells (**A**) and CD4^+^ T cells (**B**) on both tumor and FNA, but not blood. Left panel represents % positivity of inhibitory receptors for individual samples (B = blood, F = FNA, T = tumor) and right panel depicts Mean % positivity of inhibitory receptors ± SEM across dataset.

Since there is considerable evidence that pre-existing characteristics of the tumor microenvironment impact response to immuno-modulators^5^,^15^,^16^,^17^,^18^,^19^,^20^, we evaluated expression of T cell inhibitory receptors in FNA biopsies. We were able to generate data characterizing both the presence and suppressed status of CD8^+^ T cells (Fig. 3A) and CD4^+^ T cells (Fig. 3B) in the tumor microenvironment. High levels of PD-1 and moderate to low levels of TIM-3 were found on both lineages and, again, FNA data recapitulated what we observed for whole tumor. More comprehensive immunophenotyping was carried out on bulk tumor samples including measurement of CD4^+^FOXP3^+^ Tregs, myeloid derived suppressor cell subsets, and suppressed T cells, and the correlation of these populations with PD-L1 IHC (Awad *et al*. manuscript submitted). Phenotypic profiling of FNA from three NSCLC samples (Fig. 1A) for which we had complete flow annotation also exhibited consistency for CD4^+^ and CD8^+^ T cell suppressed markers when compared to bulk tumor (Supplementary Figure 7).

### Hierarchical clustering of FNA reveals distinct immunophenotypic signatures

Due to the low cell yield from FNA, we were unable to profile them with our full panel array used for bulk resected tumors (Supplementary Figure 2). However, we were able to employ two flow cytometry panels that included multiple T cell checkpoint molecules (PD-1, TIM-3, LAG-3) on anti-tumor T cell effector populations and immunosuppressive markers (PD-L1, PD-L2) on immunosuppressive myeloid subsets. Limited panels used in analysis of FNA yielded 41 parameters (Supplementary Figure 1), which we used to perform hierarchical clustering. Clustering analysis revealed that FNA samples from mesotheliomas most closely align with mesothelioma tumor samples with the exception of Patient F and, together with tumors, are well separated from normal blood samples. FNA and tumor samples are distinguished by low abundance of CD66b^+^ granulocytes, higher expression of PD-L1 and PD-L2 suppressive checkpoint co-receptors on CD33^+^ and CD66b^+^ cells, and higher expression of PD-1, TIM-3 and LAG-3 inhibitory receptors on CD3^+^ T cells (upper right quadrant of figure, yellow enrichment). Furthermore, heterogeneity between tumor samples revealed a continuum of immunosuppression and, therefore, a potential metric for assessing candidacy for immune checkpoint blockade or immunostimulation.

## Discussion

We demonstrate here that characterization of FNA biopsies by flow cytometry is a promising methodology to quantitatively assess the immune cell composition and phenotype of the bulk tumor microenvironment. Fine needle aspirates (FNA) delivered a higher fraction of viable cells than needle core biopsies (Fig. 1A) or the bulk resected tumor (Fig. 1B). The dissociation process is lengthy and may preferentially affect some cell types. For instance, we have observed that epithelial cells and some leukocytes, such as neutrophils, tend to die during the process of the dissociation (data not shown). Cells collected by FNA are already in single cell suspension, which likely accounts for their increased viability relative to dissociated core biopsies or bulk tumor. Because fewer cells are excluded, flow cytometric analysis of FNA samples may actually be more representative of the tumor microenvironment than analysis of dissociated whole tumor.

**Figure 4:**
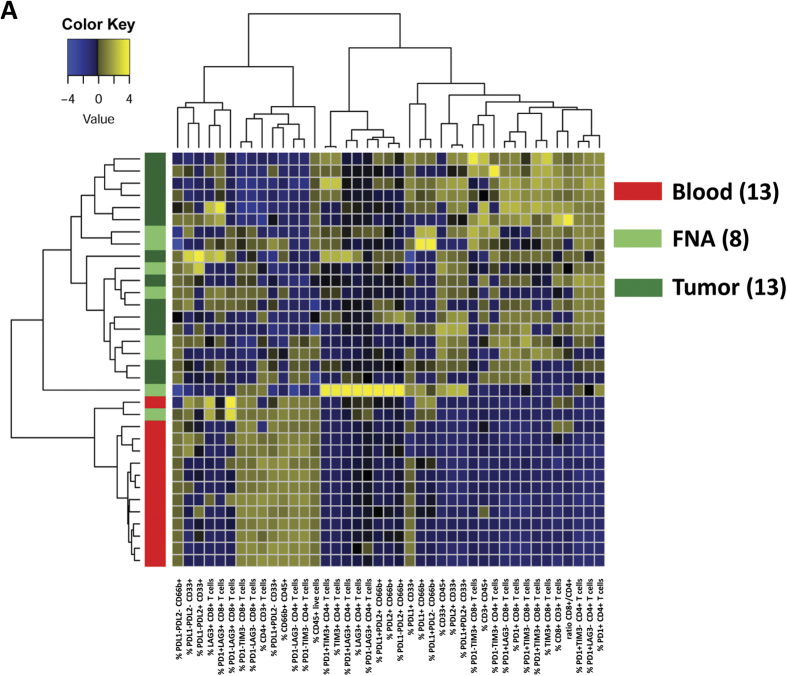
Mesothelioma FNA and mesothelioma tumors cluster together based on flow cytometry immunophenotyping. Side bar color codes of the dendrogram are such that red stands for blood samples (tissue type = 1), light green for FNA samples (tissue type = 2), and dark green (tissue type = 4) for tumors (same color scheme as in Fig. 2 t-SNE). Parameters are included below the x-axis and in Supplementary Figure 1).

We analyzed matching FNA, dissociated whole tumor, and blood for a cohort of patients (Fig. 2). Using major leukocyte lineage markers (CD3, CD4, CD8, CD66b, CD33, CD19, and CD56) we were able to determine using Ward’s minimum variance algorithm that leukocyte abundances and phenotypes determined from FNA samples largely are representative of the whole tumor. Unbiased clustering confirmed the accuracy of sample acquisition and flow cytometric analysis, as all patient blood samples clustered together while whole dissociated tumor and FNAs clustered together but were distinct from blood, with the exception of Patient F (Figs 2C and 4).

There were, however, instances in which FNA and tumors generated slightly discordant results. For instance, FNA from Patient B and Patient K had higher percentages of CD66b^+^ granulocytes relative to bulk tumors (Fig. 2). Yet the CD4^+^ and CD8^+^ T cells from these FNAs displayed similarly high expression of PD-1 and TIM-3 that was not observed in their matched blood (Fig. 3). Alternatively, there are instances such as Patient D in which the expression of PD-1 and TIM-3 by CD8^+^ T cells are reduced in FNA relative to bulk tumors (Fig. 3), but the cellular composition between FNA and bulk tumor is highly concordant and drastically different from matched blood (Fig. 2). Comparison between blood, FNA, and bulk tumor must, therefore, take into account a combination of leukocyte infiltrate and phenotypic markers. Integration of these multiple parameters by hierarchical clustering or multidimensional reduction methodologies like t-SNE enable us to discern whether FNA are or are not representative of tumor tissue. Such information will be critical for patients receiving immunotherapy as the quality of the anti-tumor immune response is predicted to depend largely on the leukocyte infiltrate and the expression of immune checkpoints by those leukocytes at time of therapy^14^.

This dataset also establishes an internal quality control component whereby freshly analyzed samples can be compared to historical data in order to determine whether FNAs more closely match the “blood signature” (Fig. 2B). For instances in which FNA samples yielded discordant results when compared to whole dissociated tumor, we feel confident concluding that the FNA procedure collected blood. FNAs are ultrasound-guided and, while sample collections tend to have high success rates, even on-target FNA can cause bleeding that results in aspiration of blood. Discordant results for Patient F (Fig. 2A) were likely due to unintended sampling of adjacent vasculature. Additionally, our data were generated using a single pass protocol, which may account for 5/13 samples yielding too few cells for analysis. Pooling of multiple passes should improve the likelihood of obtaining sufficient numbers of cells to perform flow cytometry.

Multiparametric flow cytometric analysis already enables the collection of high volumes of data for immunophenotyping, but it is possible to improve the FNA pipeline. The analysis reported in this manuscript was performed utilizing a cell analyzer with 8 channels, but we have developed assays for implementation on an instrument with the capability to simultaneously measure 19 channels. This experimental design is also compatible with CyTOF mass cytometry for analysis of over 40 proteins^21^. Sequential improvement in the analysis hardware will enable collection with more granularity that can only improve the capacity of FNAs to provide actionable data, not only regarding cell surface phenotypic markers, but intracellular signaling states as well.

The FNA immunophenotyping pipeline we have developed assesses the suppressed status of CD8^+^ and CD4^+^ T cells in the tumor microenvironment (Fig. 3). These data, when combined with the characterization of immunosuppressive markers PD-L1 and PD-L2 on CD33^+^ monocytes and CD66b^+^ granulocytes (Fig. 4) generates information as to the degree of immunosuppression present in the tumor and, hence, may predict potential response to immunomodulatory drugs. This kind of actionable predictive information is of particular interest with regard to patients that present with unresectable disease. And, as stated above, FNA data is representative of whole tumors, precluding the need for time-consuming histopathological analysis in order to determine course of treatment.

Our FNA data is collected and analyzed within 48hr of biopsy, which is valuable in the context of clinical trials as FNA can rapidly ascertain response to experimental treatments. Such data opens a window of opportunity to assess response to immunotherapeutic agents, to elucidate mechanisms of action, and can reveal potential mechanisms of acquired resistance. The most comprehensively studied immunotherapy is monoclonal antibodies that block PD-1 or PD-L1, which have been developed by multiple companies and have demonstrated clear efficacy^22^,^23^. The majority of patients, with the exception of those with Hodgkin’s lymphoma, experience no clinical response and, furthermore, many who initially respond become resistant to the therapy. Additionally, there is heterogeneous response within patients where some tumor nodules shrink while others increase in size over the course of treatment. It is not known if non-responders lack PD-1/PD-L1 expression, if their CD8^+^ T cells express compensatory checkpoint molecules like TIM-3 and CTLA-4, or if immunosuppression is mediated through alternative mechanisms like IDO or Arginase expression by myeloid-derived suppressor cells. It was hypothesized that PD-L1 expression would predict response to anti-PD-1 therapy, but patients with both PD-L1-positive and PD-L1-negative tumors respond to PD-1 blockade^14^. Whether this is due to the use of non-standardized IHC techniques in determining PD-L1 expression, or if the dynamic expression of PD-L1 and PD-1 precludes their use as predictive biomarkers, it is clear that more information is necessary to understand mechanisms of response and resistance. Routine FNA immunophenotypic profiling may provide this crucial data.

Fine needle aspirate biopsies routinely collect enough viable cells for flow cytometric immunophenotyping with multiple antibody panels and yield data that is representative of the whole tumor. Interrogation of numerous immune cell lineages and inhibitory and immunosuppressive markers allowed us to discern the state of immunosuppression in the tumor microenvironment. Such minimally invasive longitudinal phenotyping could become an essential component of clinical trials in order to determine response to immunomodulatory agents or as a rationale to enroll patients for treatment with such therapies. Retrospective analysis of datasets may reveal better predictive biomarkers to stratify responders and non-responders.

## Methods

### Tumor preparation, flow cytometry, and antibodies

Fresh tissue was minced in a 10cm dish then resuspended in dissociation buffer consisting of RPMI (Life Technologies, Carlsbad, CA) +10% FBS (HyClone, Logan, UT), 100 U/mL collagenase type IV (Life Technologies, Carlsbad, CA), and 50 μg/mL DNase I (Roche, Indianapolis, IN) at a ratio of 5 mL of dissociation buffer per 500 mg of sample. Suspension was incubated at 37 °C for 45–50 min then further dissociated by passing through a syringe. Red blood cells were removed from samples using red blood cell lysis buffer (BioLegend, San Diego, CA). Samples were pelleted and then resuspended in fresh RPMI +10% FBS and strained over a 70 μm filter. Cells were incubated for 8 min in the dark at room temperature with Live/Dead Fixable Yellow Dead Cell Stain Kit (Life Technologies, Carlsbad, CA) in FACS buffer (PBS +2% FBS) at a ratio of 250 μL L/D 1X dilution per 100 mg of original sample weight. Surface marker and intracellular staining were performed according to the manufacturer’s protocol (eBioscience, San Diego, CA). FcR were blocked prior to surface antibody staining using Human FcR Blocking Reagent (Miltenyi, San Diego, CA). Cells were fixed in 1% formalin in PBS +2% FBS and washed prior to analysis on a BD FACSCanto II HTS cell analyzer with FACSDiva software (BD Biosciences, San Jose, CA). Data were analyzed using FlowJo (Ashland, OR) software version 10.0.8. Cell viability was determined by negative live/dead staining. Antibodies were specific for the following human markers: CD3 (HIT3a; UCHT1), CD8 (RPA-T8), CD14 (M5E2; MphiP9), CD45 (HI30), CD56 (B159), CD279 (EH12.1), HLA-DR (G46-6), and IgG1 isotype control (MOPC-21) from BD Biosciences (San Jose, CA); CD4 (RPA-T4), CD16 (3G8), CD19 (HIB19), CD33 (WM53), CD66b (G10F5), CD123 (6H6), CD273 (24F.10C12), CD274 (29E.2A3), CD326 (9C4), TIM3 (F38-2E2), CTLA-4 (L3D10), IgG2a isotype control (MOPC-173), IgG2b isotype control (MPC-11), and IgG1 isotype control (MOPC-21) from BioLegend (San Diego, CA); CD45 (2D1) and FOXP3 (236A/E7) from eBioscience (San Diego, CA); and LAG3 (polyclonal) and isotype control (polyclonal) from R&D Systems (Minneapolis, MN).

### Collection of fine needle aspirates

FNA and core needle biopsy specimens of non-small cell lung cancer tumors were obtained *ex vivo* from freshly resected specimens. All experimental protocols were approved by DF-HCC protocol 98-063 and all were performed in accordance with relevant guidelines and regulations. Written informed consent was obtained from all subjects. For *in vivo* FNA, subjects were patients with suspected or proven malignant pleural mesothelioma who had enrolled with informed consent on Dana-Farber/Harvard Cancer Center (DF/HCC) protocol 04-349. Once plans for the appropriate surgical procedure were confirmed, a study radiologist reviewed the preoperative chest CT or MRI to identify the best area for biopsy that is near the future skin incision line and confirmed that the thickness of the pleura/tumor was adequate. Once the patient was in the operating room and anesthesia induction had taken place, the study surgeon used a hand held operative ultrasound assisted by a radiologist to guide a 22 gauge, 2 inch long spinal needle into the pleural tumor in the appropriate location. The needle had been marked for depth that was determined pre-operatively based on the CT. The aspirate was then ejected into 1 mL of sterile medium (DMEM +FBS). This procedure was performed prior to surgical incision that is part of the scheduled procedure. Portions of the resected tumor, uninvolved lung tissue, and a control sample consisting of 200 μL anticoagulated whole blood ejected into a separate 1 mL aliquot of sterile medium, were also obtained with informed consent under DF/HCC protocol 98-063 when available.

### Data analysis

Data were visualized using bar graphs and compared using the unpaired Student *t* test. Error bars represent standard error of the mean from independent samples analyzed under the indicated parameters. The following denote statistical significance: *p-value < 0.05; **p-value < 0.01; ***p-value < 0.001. In order to classify the FNA and tumor samples in comparison with the whole blood, we applied the Hierarchical Agglomerative Clustering method^24^ using the Ward’s minimum variance algorithm to the 34 (13 blood, 8 FNAs, 13 mesotheliomas) samples’ 41 immune profile parameters (Supplementary Figure 1). Ward algorithm aims at finding compact spherical clusters so that the clusters bear minimum variance within themselves. The data was first Z-score normalized and then clustered with the ward.D2 approach^25^ implemented in R.3.2.2 and visualized with the heatmap.2 function in R.gplots package. In addition, unsupervised non-linear dimension reduction method t-SNE^26^ was applied to investigate in reduced dimension space how samples from all 8 patients that have complete blood, FNA, and tumor flow data are located in relation to each other. t-SNE denotes t-distribution based stochastic non-linear embedding method, which minimizes the divergence of neighborhood closeness moving from high dimensions to low dimensions. For the embedding, 22 parameters included 1–11 and 19–29 (Supplementary Figure 1).

## Additional Information

**How to cite this article**: Lizotte, P. H. *et al*. Fine needle aspirate flow cytometric phenotyping characterizes immunosuppressive nature of the mesothelioma microenvironment. *Sci. Rep.* **6**, 31745; doi: 10.1038/srep31745 (2016).

## Supplementary Material

Supplementary Information
