# Supplementary material for: Fine needle aspirate flow cytometric phenotyping characterizes immunosuppressive nature of the mesothelioma microenvironment
Source: Sci Rep. 2016 Aug 19;6:31745. doi: 10.1038/srep31745 (PMC4990967; doi:10.1038/srep31745)
Supplement: Supplementary Information [file srep31745-s1.pdf]

Fine needle aspirate flow cytometric phenotyping characterizes immunosuppressive nature of the mesothelioma microenvironment

Patrick H Lizotte[1,2], Robert E Jones[1,2], Lauren Keogh[1,2], Elena Ivanova[1,2], Hongye Liu[1,2], Mark M Awad[2,3,7], Peter S Hammerman[2,3,7], Ritu R Gill[2,4], William G Richards[5,6,7], David A Barbie[2,7], Adam J Bass[2,7], Raphael Bueno[5,6,7], Jessie M. English[1,2], Mark Bittinger[1,2], Kwok-Kin Wong[1,2,3,7,*]

[1]Belfer Center for Applied Cancer Science 360 Longwood Ave. Boston, MA 02115

[2]Department of Medical Oncology and [3]Lowe Center for Thoracic Oncology

Dana-Farber Cancer Institute 450 Brookline Ave Boston, MA 02115

[4]Department of Radiology, [5]Division of Thoracic Surgery, [6]International Mesothelioma Program of the Lung Center Surgery at Brigham and Women's Hospital 75 Francis St. Boston, MA 02115

[7]Harvard Medical School 25 Shattuck St, Boston, MA 02115

*Corresponding author: Kwok-Kin Wong, Dana-Farber Cancer Institute 450 Brookline Avenue Boston, MA 02215, phone 617-632-6084, fax 617-582-7683, e-mail kwok-kin_wong@dfci.harvard.edu

Financial support: Robert A. and Renée E. Belfer Foundation, Expect Miracles Foundation, Starr Consortium for Cancer Research, the Damon Runyon Cancer Research Foundation, and Stand Up 2 Cancer Foundation (SU2C).

[1] %CD45+
[2] %CD3+ T cells
[3] %CD33+ Monocytes
[4] %CD66b+ Granulocytes
[5] %CD4 T cells
[6] %CD8 T cells
[7] CD8 vs CD4 ratio
[8] % CD8 TIM3-PD1+
[9] % CD8 TIM3+PD-1+
[10] % CD8 TIM3+PD-1-
[11] % CD8 TIM3-PD-1-
[12] % CD8 LAG3-PD-1+
[13] % CD8 LAG3+PD-1+
[14] % CD8 LAG3+PD-1-
[15] % CD8 LAG3-PD-1-
[16] % CD8 TIM-3
[17] % CD8 PD-1
[18] % CD8 LAG-3
[19] % CD4 TIM3-PD1+
[20] % CD4 TIM3+PD-1+
[21] % CD4 TIM3+PD-1-
[22] % CD4 TIM3-PD-1-
[23] % CD4 LAG3-PD-1+
[24] % CD4 LAG3+PD-1+
[25] % CD4 LAG3+PD-1-
[26] % CD4 LAG3-PD-1-
[27] % CD4 TIM-3
[28] % CD4 PD-1
[29] % CD4 LAG-3
[30] %CD33 PD-L2-PD-L1+
[31] % CD33 PD-L2+PD-L1+
[32] % CD33 PD-L2+PD-L1-
[33] % CD33 PD-L2-PD-L1-
[34] % CD33 PD-L1
[35] % CD33 PD-L2
[36] %CD66b PD-L2-PD-L1+
[37] % CD66b PD-L2+PD-L1+
[38] % CD66b PD-L2+PD-L1-
[39] % CD66b PD-L2-PD-L1-
[40] % CD66b PD-L1
[41] % CD66b PD-L2

Supplementary Figure 1. List of 41 parameters used for hierarchical clustering in Figure 4. A subset of these was also used for tSNE in Figure 2.

A

		Name	ID	AF488	PE	PerCp-Cy5.5	APC	PacBlue	PE-Cy7	APC-H7
				Fluorochrome						
Immune Analysis	Surface	Lympho*	1	CD56	**CD8**	CD16	**CD3**	CD45	CD19	CD4
		T1*	2	CD4	CD38	**HLA-DR**	CD45RA	CCR7	CD3	CD8
		T2	3	CD45	Tim-3	CD8	**LAG3**	CD3	PD-1	CD4
		T2 iso	4	CD45	iso	CD8	**iso**	CD3	iso	CD4
		T3	5	CD4	Tim3	CD8	CD45RO	CD3	PD-1	CD45RA
		T4	6	**CD11a**	**CD69**	CD8	CXCR3	CD3	PD-1	CD4
		Myelo*	7	CD45	CD66b	**HLA-DR**	CD33	CD14	CD123	CD16
		Tumor*	8	CD45	PD-L2	CD66b	PD-L1	CD33	EpCAM	CD14
		Tumor iso*	9	CD45	iso	CD66b	iso (PD-L1)	CD33	EpCAM	CD14
	Intra	T1	10	CD45	CTLA-4	CD8	FOXP3	CD3	PD-1	CD4
		T2	11	CD45	iso	CD8	FOXP3	CD3	iso	CD4
		T3	12	CD45	Tim-3	CD8	FOXP3	CD3	Ki67	CD4
Sorting			1	CD45	CD56	CD8	CD33	EpCAM	CD4	CD16

B

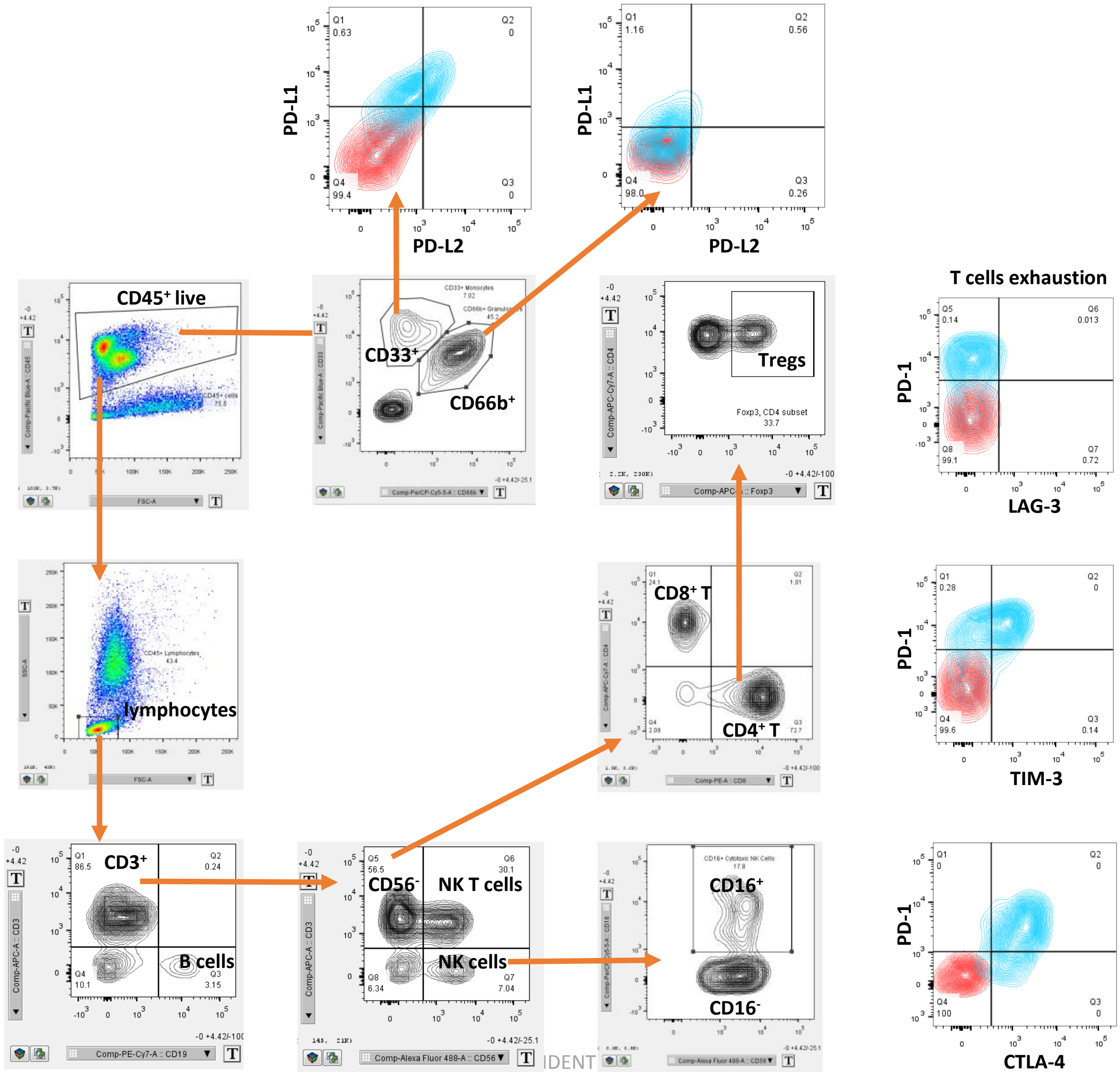

Supplementary Figure 2. A, Table of antibody panels with surface markers (black) and intracellular markers (red). B, Flow cytometry gating progression based on lineage markers. Lymphocytes and myeloid cells are assayed for expression of immunomodulatory phenotypic markers.

A

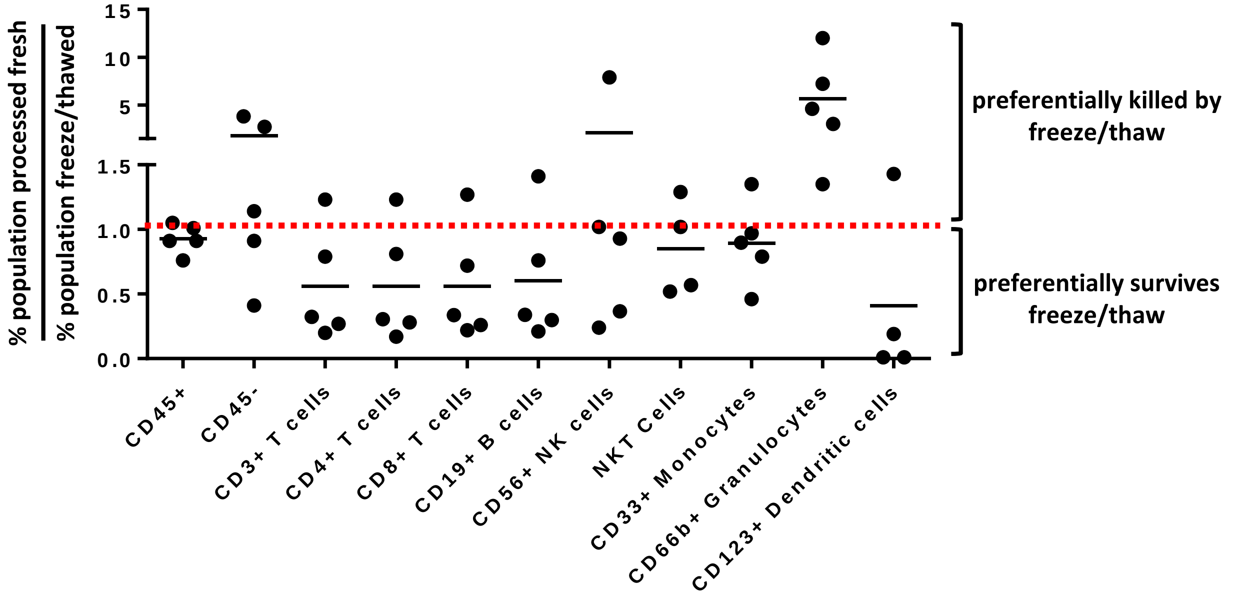

Supplementary Figure 3. Freeze/thaw preferentially kills CD45- cells and granulocytes while lymphocytic population proportions are unaffected. Tumor samples included one pleural effusion, one NSCLC, and three mesotheliomas.

patient	sample type	live cells per sample
	peripheral blood	130,000
A	FNA	73,000
	resected tumor	194,000
	peripheral blood	95,000
B	FNA	18,000
	resected tumor	220,000
	peripheral blood	112,000
D	FNA	2,400
	resected tumor	300,000
	peripheral blood	64,000
F	FNA	27,000
	resected tumor	43,000
	peripheral blood	69,000
H	FNA	78,000
	resected tumor	68,000
	peripheral blood	57,000
J	FNA	1,200
	resected tumor	130,000
	peripheral blood	51,000
K	FNA	44,000
	resected tumor	700,000
	peripheral blood	65,000
M	FNA	900
	resected tumor	7,000
	peripheral blood	80,375
average	FNA	30,563
	resected tumor	207,750

Supplementary Figure 4. List of live cells run by flow cytometry from blood, bulk resected tumor, and FNA biopsy from 8/13 mesothelioma patients whose FNA yielded sufficient cells for analysis.

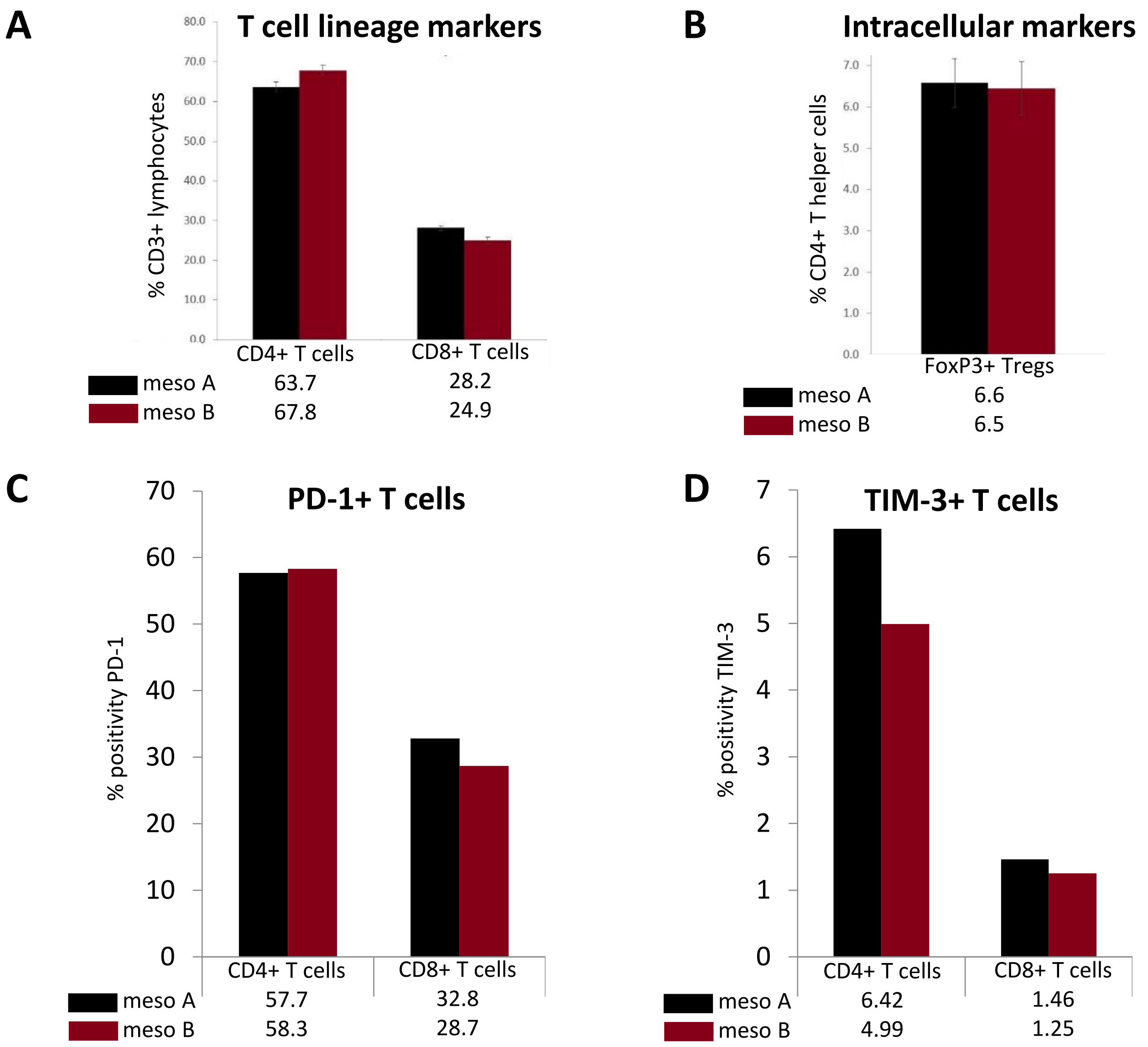

Supplementary Figure 5. Flow cytometric profiling of mesothelioma immune microenvironment is highly accurate and reproducible. A-B, Tissue resection was received in two large pieces and processed separately: dissociation, live/dead staining, FcR block, surface staining (A), and intracellular staining (B) were carried out in parallel. Technical replicates exhibit concordance in % positive surface antigen and intracellular transcription factor staining. C-D, Lymphocytes also show high degree of similarity in expression of phenotypic markers (exhaustion markers PD-1 and TIM-3) between replicates.

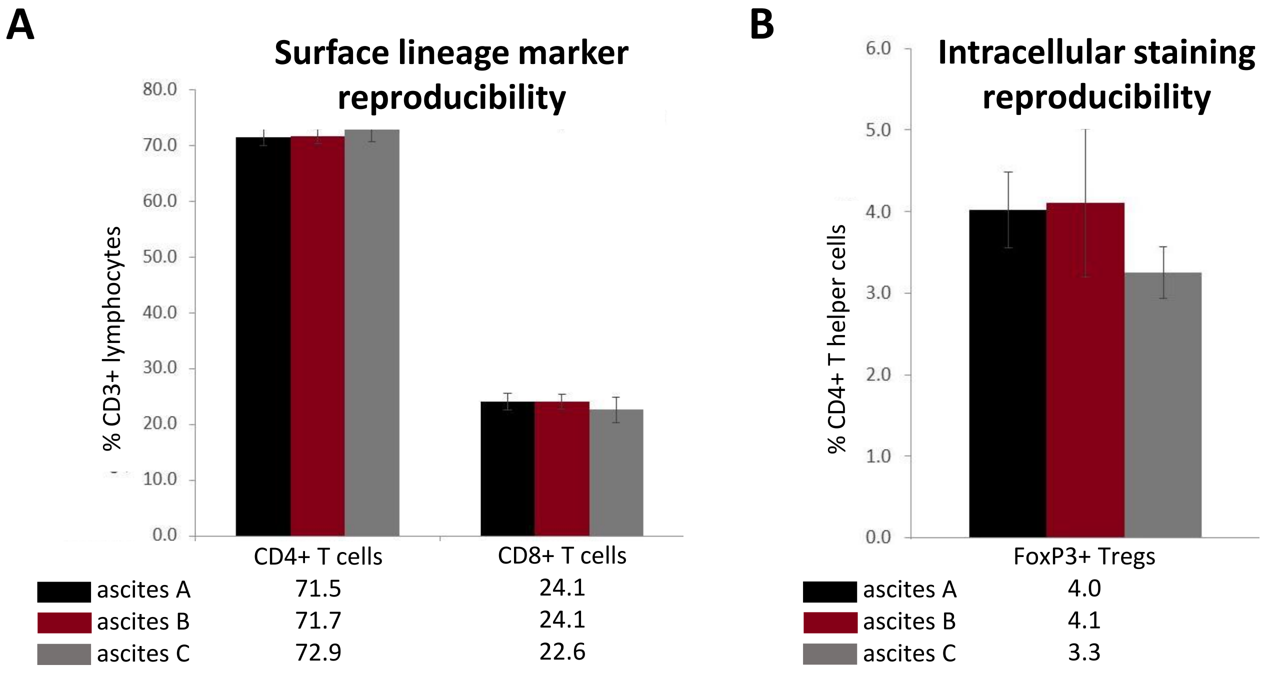

Supplementary Figure 6. Flow cytometric immunophenotyping exhibits consistency with variable cell numbers. A, Ascites fluid from a gastro-instestinal tumor was analyzed by flow cytometry. “Ascites A” and “ascites B” are technical replicates, whereas “ascites C” is a 1:20 cell dilution. Minimal variability is observed even with varying numbers of cells. B, Same experimental design was also assayed for presence of FoxP3+ T-regs. Even at low cell numbers (ascites C) we were able to measure a comparable percentage of T-regs out of the CD4+ T helper cell compartment.

A

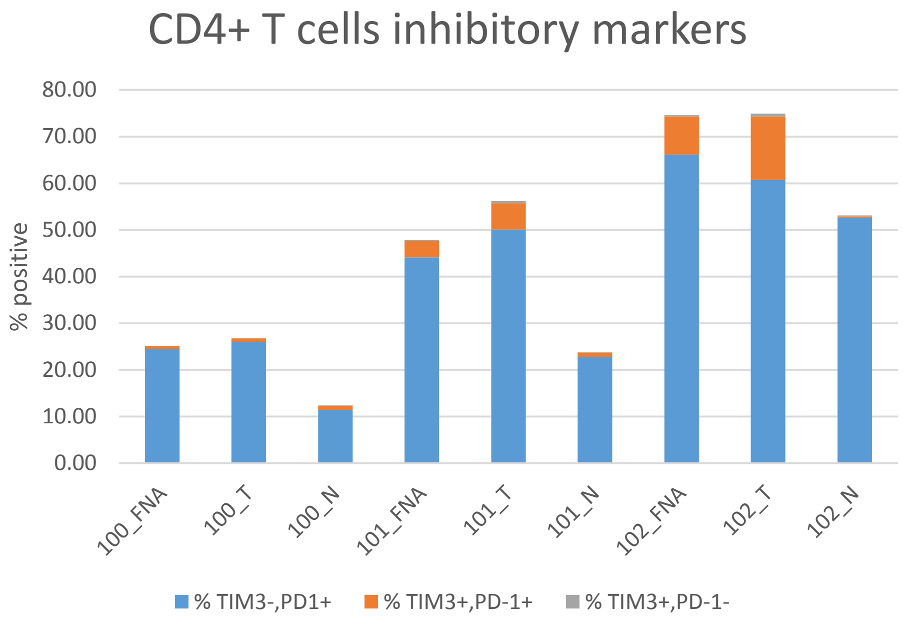

B

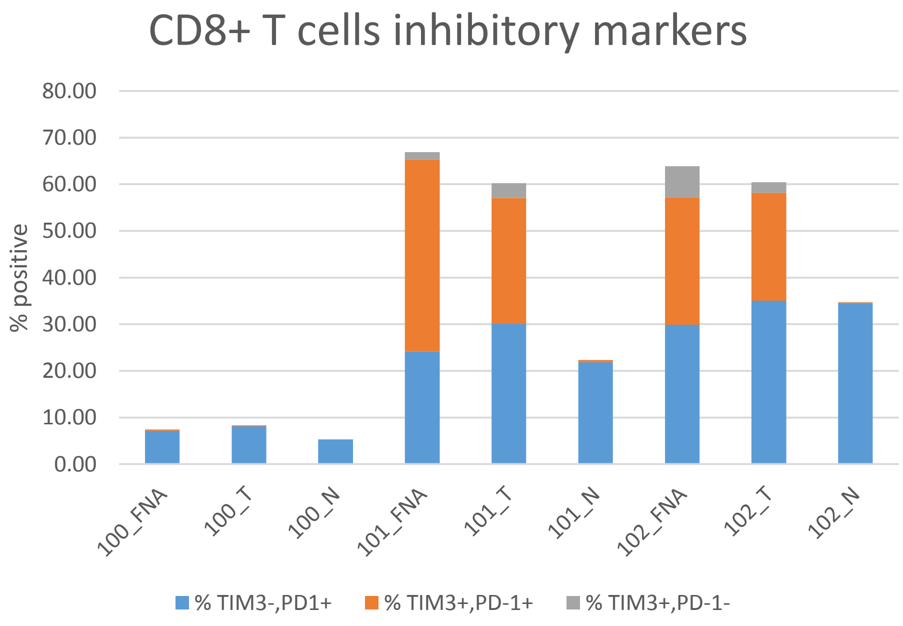

Supplementary Figure 7. Analysis of NSCLC FNA by flow cytometry characterizes tumor immunophenotype. A-B, Immunoprofiling revealed high expression of exhaustion markers on CD4+ T cells (A) and CD8+ T cells (B) on both tumor and FNA, but not normal lung tissue.

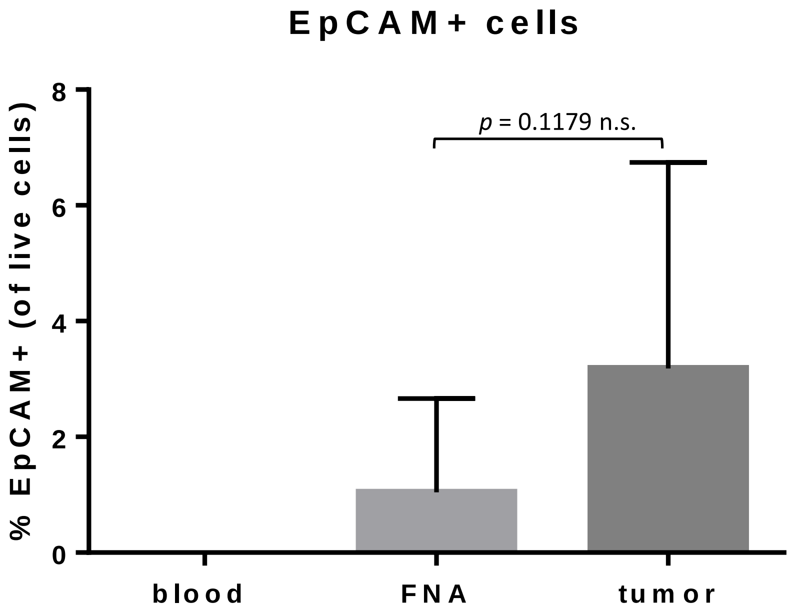

Supplementary Figure 8. Analysis of % EpCAM+ cells in mesothelioma FNA (cases A, B, D, F, H, J, K, M) from Figure 2 by flow cytometry. Blood samples yielded zero EpCAM+ cells vs. 1.043 ± 0.6129 (mean ± SEM) for FNA vs. 3.186 ± 1.345 (mean ± SEM) for bulk tumor. Data was analyzed using a paired *t*-test and there was no observed significant difference between FNA and bulk tumor.
